# Cross-linking of Phospholipid Membranes is a Conserved Property of Calcium-sensitive Synaptotagmins

**DOI:** 10.1016/j.jmb.2008.01.084

**Published:** 2008-06-27

**Authors:** Emma Connell, Asiya Giniatullina, Joséphine Lai-Kee-Him, Richard Tavare, Enrico Ferrari, Alan Roseman, Dan Cojoc, Alain R. Brisson, Bazbek Davletov

**Affiliations:** 1 MRC Laboratory of Molecular Biology, Hills Road, Cambridge CB2 0QH, UK; 2 CNR-INFM Laboratorio Nazionale TASC, 34012 Trieste, Italy; 3 IECB, UMR-CNRS 5248, University of Bordeaux, F-33405 Talence, France; 4 Faculty of Life Sciences, University of Manchester, Manchester, UK

**Keywords:** C2 domain, membrane cross-linking, synaptotagmin, electron microscopy, laser trap

## Abstract

Synaptotagmins are vesicular proteins implicated in many membrane trafficking events. They are highly conserved in evolution and the mammalian family contains 16 isoforms. We now show that the tandem C2 domains of several calcium-sensitive synaptotagmin isoforms tested, including *Drosophila* synaptotagmin, rapidly cross-link phospholipid membranes. In contrast to the tandem structure, individual C2 domains failed to trigger membrane cross-linking in several novel assays. Large-scale liposomal aggregation driven by tandem C2 domains in response to calcium was confirmed by the following techniques: turbidity assay, dynamic light-scattering and both confocal and negative stain electron microscopy. Firm cross-linking of membranes was evident from laser trap experiments. High-resolution cryo-electron microscopy revealed that membrane cross-linking by tandem C2 domains results in a constant distance of ∼9 nm between the apposed membranes. Our findings show the conserved nature of this important property of synaptotagmin, demonstrate the significance of the tandem C2 domain structure and provide a plausible explanation for the accelerating effect of synaptotagmins on membrane fusion.

## Introduction

Intracellular membrane traffic is governed by a set of conserved proteins that includes members of the synaptotagmin family.^1,2^ Synaptotagmins are membrane proteins that possess tandem C2 domains (also known as C2AB) implicated in calcium-dependent phospholipid binding.^3^ Calcium-sensitive synaptotagmins are thought to confer calcium-sensitivity to the fusion of secretory vesicles with target membranes. In particular, brain synaptotagmin I has an important role in coupling the calcium signal to the release of neurotransmitters in neurons and is strikingly conserved in metazoan evolution.^4,5^ Knockout of the synaptotagmin 1 gene in mouse and *Drosophila* model organisms results in a lethal phenotype.^5,6^ The 16 identified synaptotagmins are expressed at different levels in various tissues and tag specific vesicles contributing to the diversity of intracellular membrane traffic.^7,8^ Some isoforms are not calcium-sensitive and have been implicated in alternative cellular processes including vesicle maturation.^9,10^ All synaptotagmins share the following organization: a single transmembrane region connected through a variable-length linker to two closely spaced (i.e., tandem) C2 domains. Structurally, a C2 domain consists of eight beta-strands arranged in a barrel-like structure with a total length of 125–130 amino acids.^11^ C2 domains were initially identified in the protein kinase C (PKC) family and are found in more than 100 intracellular proteins, including many membrane-trafficking proteins.^12^

A wealth of data is now available on the binding of synaptotagmin C2 domains to a single phospholipid membrane, and on their interactions with other proteins involved in membrane fusion.^13–15^ Intriguingly, an early study of synaptotagmin 1 demonstrated that tandem C2 domains can aggregate chromaffin granules,^16^ whereas Arac and colleagues reported recently that even a single C2 domain is sufficient for cross-linking of liposomal membranes.^17^ Since membrane cross-linking may have an important role in membrane fusion, we decided to investigate different synaptotagmin isoforms and the C2 domain requirement for this process using several alternative techniques. Our data indicate that the tandem C2 domain structure is necessary for membrane apposition and that this tandem C2 domain cross-linking property is well conserved in the synaptotagmin family, correlating well with calcium-phospholipid binding ability. Cryo-electron microscopy revealed that tandem C2 domains trigger membrane apposition at a defined distance of ∼ 9 nm.

## Results

**Fig. 1:**
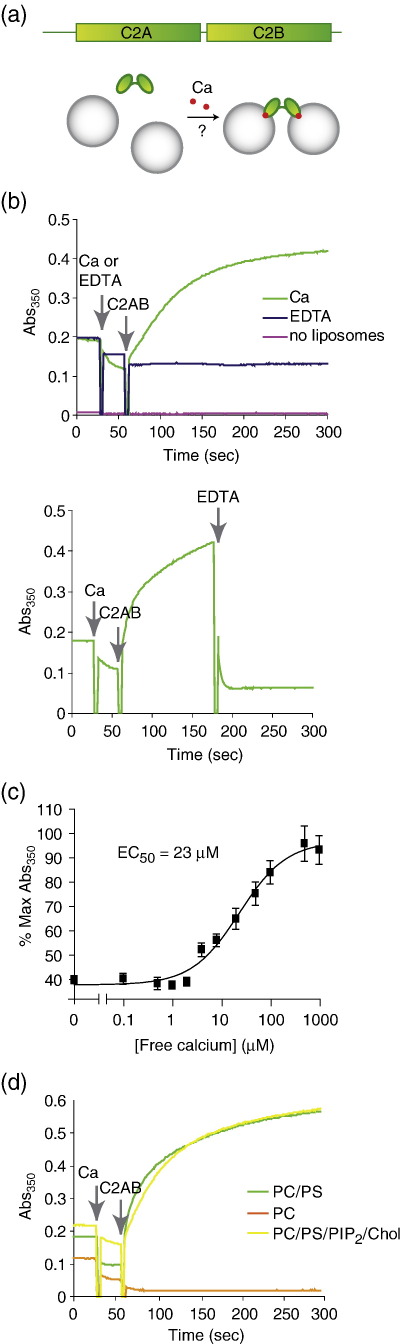
Synaptotagmin 1 C2AB promotes membrane cross-linking efficiently. Liposomal aggregation was assessed by measuring changes in solution absorbance (turbidity) at 350 nm upon addition of calcium and/or protein. Protein (2.5 μM) was added to 0.5 mg/ml liposomes made of phosphatidylcholine/phosphatidylserine (PC/PS, 3:1 molar ratio). (a) Schematic showing the tandem C2 domain structure of the cytosolic part of synaptoagmins, C2AB, and the hypothetical mode of calcium-dependent liposomal cross-linking. (b) Top; C2AB aggregates liposomes in the presence of 1 mM free calcium but not EDTA. No turbidity change was were seen in the absence of liposomes. Bottom; the liposomal cross-linking by C2AB/calcium is reversible upon addition of EDTA (final concentration 2 mM). The small reductions in turbidity immediately after addition of each reagent reflect 20% dilution of the reaction mixture. (c) Calcium titrations give an EC_50_ value of 23 μM for calcium-dependent C2AB-mediated liposome aggregation. Absorbance is expressed as a percentage of the maximum; the data shown are the results of three independent experiments. (d) Phosphatidylserine, PS, is an essential cofactor for C2AB-mediated liposomal aggregation. A complex lipid ratio of PC/PS/PIP_2_/cholesterol (60:25:5:10) produced similar results to PC/PS alone. PIP_2_, phosphatidylinositol 4,5-bis-phosphate.

To analyse membrane cross-linking (Fig. 1a), we adapted a simple technique used previously in annexin studies.^18^ Unilamellar liposomes of ∼80 nm in diameter were prepared by extrusion through a polycarbonate filter,^19^ and the turbidity of the liposomal solution was recorded at a wavelength of 350 nm.

Figure 1b (top panel) shows that addition of the tandem C2 domain of synaptotagmin 1 (indicated as C2AB) to liposomal solution, in the presence of calcium, led to an immediate rise in liposome aggregation. No aggregation was observed in the presence of the calcium chelator EDTA, or in the absence of liposomes. C2AB-driven liposomal aggregation was rapidly reversed upon addition of EDTA later in the reaction, indicating that C2AB can drive apposition of membranes but not their fusion (Fig. 1b, bottom panel). We carried out calcium titrations with C2AB and determined that membrane aggregation occurs with an EC_50_ of 23 μM (Fig. 1c). This value is consistent with the concentrations of calcium required for phospholipid binding,^20,21^ suggesting that membrane association and cross-linking by C2AB occur concomitantly upon a rise in local calcium concentration. Liposomal aggregation by C2AB also required the presence of phosphatidylserine, which is known to be important for calcium/phospholipid binding by each of the two C2 domains (Fig. 1d).^22–24^

**Fig. 2:**
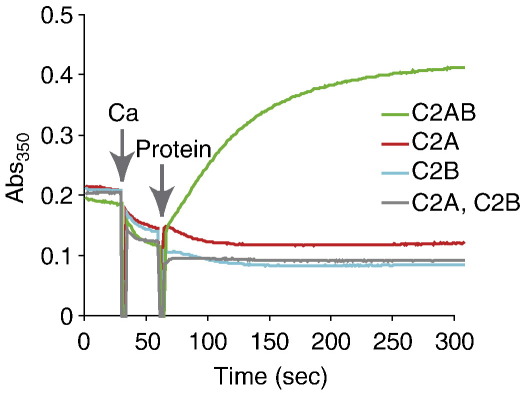
The synaptotagmin 1 tandem C2 domain structure is essential for membrane cross-linking. The tandem C2 domain structure, C2AB, is capable of promoting liposomal aggregation, whereas individual C2A, C2B, or C2A added with C2B are ineffective. Reaction conditions as for Fig. 1.

Having confirmed that C2AB is an efficient cross-linker of phospholipid membranes, we turned our attention to the individual C2 domains, C2A and C2B. Fig. 2 shows that these domains, neither alone nor added together, were capable of causing liposomal aggregation as measured by turbidity, despite the fact that each one bound in a calcium-dependent manner to phospholipid membranes in a liposome pelleting assay (Supplementary Data Fig. 1).

**Fig. 3:**
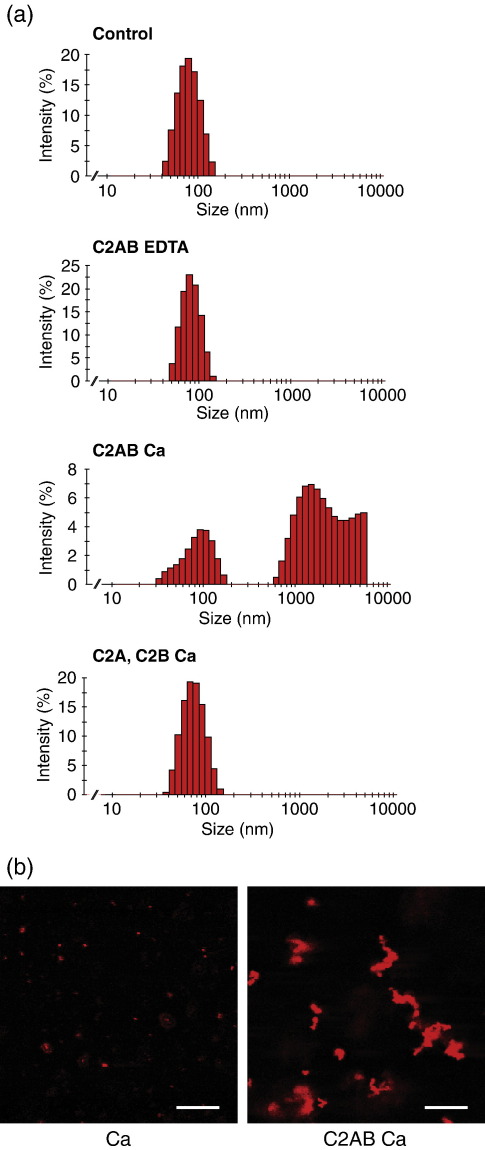
Characterisation of liposome aggregation by synaptotagmin 1 C2AB. (a) Dynamic light-scattering measurements were carried out under control conditions (no protein, no calcium), with PC/PS liposomes. In the presence of EDTA, C2AB produced no increase in particle size. In the presence of calcium, C2AB addition promoted large-scale liposomal aggregation. Addition of isolated C2A and C2B domains produced no size increase. Reactions were as in Fig. 1, and measurements were made after incubation for 5 min. (b) Confocal microscopy of DiI-labelled large unilamellar vesicles after incubation for 10 min with 1 mM calcium in the presence (right) or in the absence (left) of C2AB. The scale bar represents 10 μm.

To test C2AB function further, we employed conventional dynamic light-scattering to follow liposomal aggregation. Measurement of the liposomal size in the absence of protein or calcium alone (Fig. 3a and not shown) gave an identical unimodal particle size distribution with an average diameter of 80 nm. The particle size underwent a dramatic increase when both C2AB and calcium were present together. However, the addition of individual C2 domains, alone or together, again had no cross-linking effect (Fig. 3a and not shown).

In addition to the two techniques used above, we decided to directly observe liposomal aggregation driven by tandem C2 domains. We prepared large unilamellar vesicles, labelled with the hydrophobic dye DiI, by extrusion through a 600 nm pore size filter. Confocal microscopy of a control sample (1 mM calcium in the absence of C2AB) revealed small red particles moving freely in solution (Fig. 3b, left-hand panel). Upon addition of C2AB, very large red aggregates appeared after 5–10 min of incubation (Fig. 3b, right-hand panel), demonstrating that liposomal cross-linking had occurred. Neither C2AB in the absence of calcium, nor individual C2 domains in the presence of calcium were capable of triggering liposomal aggregation even after 60 min (data not shown). Further, we employed laser trap experiments in which a number of liposomes can be captured in a thin line along the axis of the laser beam. We observed that upon switching off the laser, in control conditions (C2AB, no calcium) the aligned liposomes immediately moved away from each other; in contrast, the addition of calcium was sufficient to bond these liposomes firmly in a caterpillar-like structure (Supplementary Data Fig. 2 and movies).

**Fig. 4:**
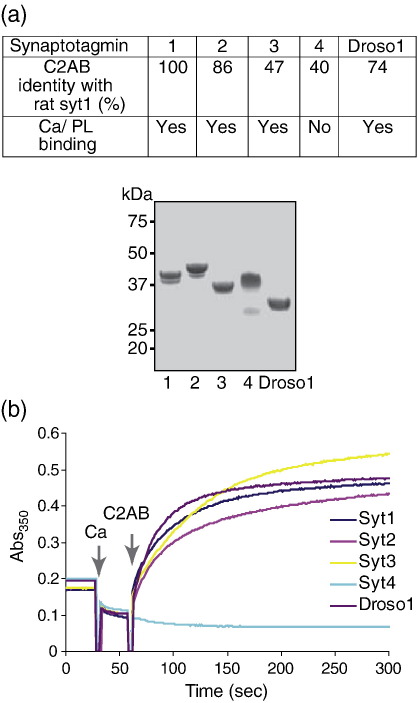
Membrane cross-linking is a conserved property of calcium-sensitive synaptogmins. (a) Top; Table indicating the degree of sequence identity between the C2AB domains of rat synaptotagmins 1–4 (Syt) and *Drosophila* synaptotagmin 1 (Droso1). Ability to bind phospholipid membranes in the presence of calcium is indicated. Bottom; Coomassie brilliant blue-stained polyacrylamide/SDS gel showing 3 μg of purified synaptotagmin isoforms. Molecular mass markers are indicated at the left. (b) A graph showing turbidity changes of liposomal solutions in the presence of indicated C2ABs and 1 mM calcium. Note, all tested calcium-sensitive synaptotagmin C2ABs were able to aggregate liposomes.

Mammalian synaptotagmins constitute a large protein family with individual members being involved in many aspects of membrane traffic, including neuronal exocytosis, vesicle maturation, plasma membrane repair, lysosomal fusion, neurite outgrowth and the sperm acrosome reaction during fertilization.^8,10,25–27^ It was thus important to investigate how well membrane cross-linking is conserved among different synaptotagmin isoforms, which differ in primary sequence by as much as 60% (Fig. 4a). Tandem C2 domains of rat synaptotagmin isoforms 1 to 4 and *Drosophila* synaptotagmin 1 were expressed and purified in parallel. The apparent molecular mass estimated by SDS-PAGE was slightly different among the synaptotagmin C2ABs but was within the range 33–40 kDa (Fig. 4a). Among these isoforms, rat synaptotagmin 4 is known to be incapable of binding phospholipid membranes.^9,28^ Our turbidity tests demonstrated that all calcium-sensitive isoforms, i.e., rat isoforms 1–3 and *Drosophila* synaptotagmin 1, were capable of liposomal cross-linking (Fig. 4b), whereas synaptotagmin 4, as expected, failed to cross-link membranes.

**Fig. 5:**
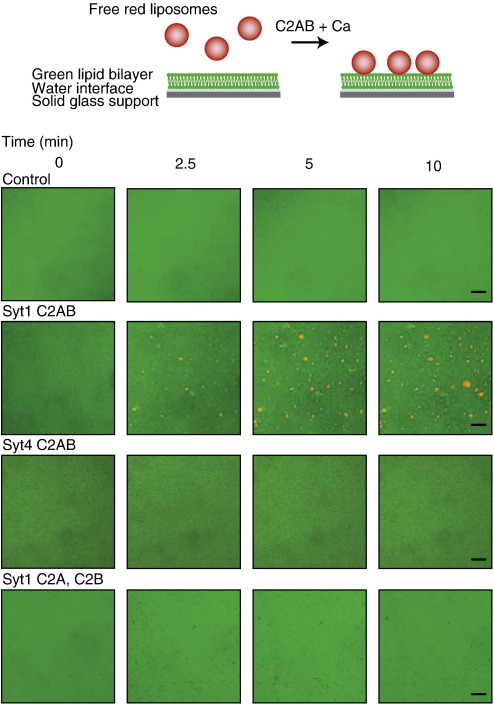
C2AB-mediated attachment of large unilamellar vesicles to a planar lipid bilayer. Top; a representation of the DiO-labelled green liposomes fused onto a flat glass surface. Free DiI-labelled red vesicles (600 nm) were added in the presence or in the absence of protein and calcium. Bottom; visualisation of vesicle docking by confocal microscopy. Red vesicles and green bilayers were incubated under control conditions (1 mM calcium alone), or with calcium plus synaptotagmin 1 C2AB, or synaptotagmin 4 C2AB or isolated synaptotagmin 1 C2A and C2B (2.5 μM each). Only synaptotagmin 1 C2AB promoted rapid docking of the red vesicles onto the bilayer. The scale bar represents 3 μm.

Next, we investigated membrane cross-linking in a new system that resembles docking of vesicles to a planar plasma membrane. For this, 80 nm liposomes were labelled green by incorporating the hydrophobic dye DiO and then allowed to fuse onto glass, producing a planar bilayer. After removal of unattached green liposomes by washing, red-labelled liposomes of 600 nm size were added in the presence or in the absence of calcium and various synaptotagmins. The behaviour of red liposomes was analysed at the level of the planar phospholipid bilayer by confocal microscopy. Tandem C2ABs of synaptotagmins 1–3 were all capable of attaching red liposomes onto the green bilayer (Fig. 5 and data not shown). These docked liposomes were firmly immobilised at the bilayer throughout our 30 min observation period (Supplementary Data Fig. 3). As expected, neither synaptotagmin 4 nor individual C2 domains of synaptotagmin 1 were able to cause vesicle ”docking” in this new assay.

**Fig. 6:**
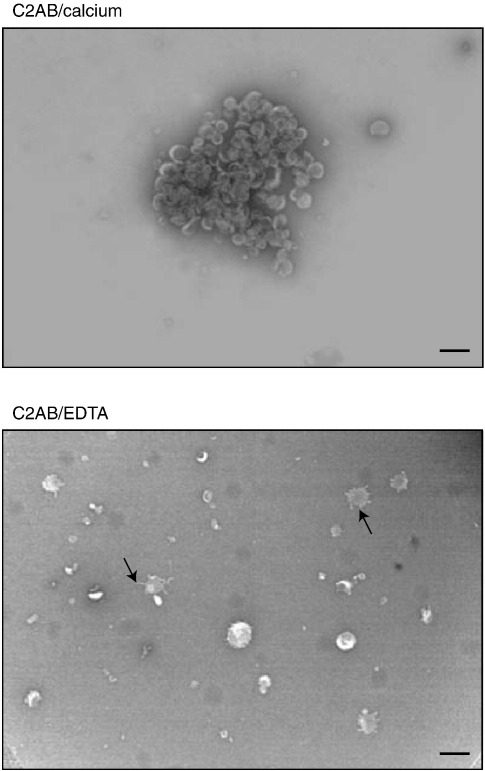
Negative-stain electron microscopy shows calcium-dependent C2AB-mediated aggregation of liposomes. Top; representative aggregate of liposomes formed in the presence of 1 mM calcium and 10 μM C2AB. Bottom; liposomes in the presence of C2AB but in the absence of calcium do not aggregate. Arrows indicate some ”splattering” of liposomes on the carbon-coated grid after staining with uranyl acetate. The scale bar represents 200 nm.

The increase in particle size with C2AB/calcium that we observed with PC/PS liposomes (Figs. 1–4) could be, in principle, due to either the formation of liposomal aggregates^16^ or large-scale membrane deformations such as tubulation.^29^ To investigate the nature of the increased particle size, we carried out negative-stain electron microscopy. Figure 6 (top panel) shows that C2AB of synaptotagmin 1 drives bulk liposome aggregation in a calcium-dependent manner. In the absence of calcium and/or C2AB, individual liposomes were well separated and occasionally we could detect small changes in liposomal shape, manifested as a ”splattered” appearance (Fig. 6, lower panel).

**Fig. 7:**
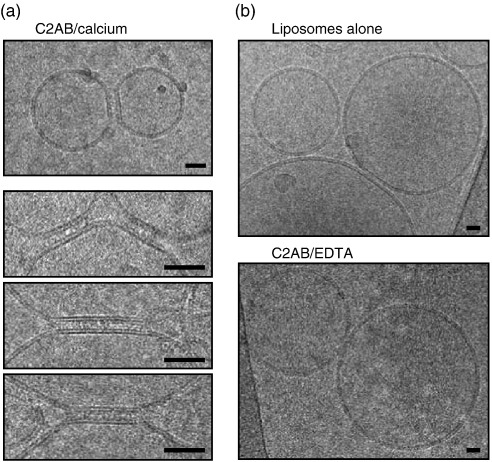
Cryo-electron microscopy indicates close apposition of two phospholipid membranes by synaptotagmin 1 C2AB. (a) C2AB (0.1 μM) in the presence of 1 mM calcium promotes formation of flat junctions between membranes. A gallery of typical junctions embedded in a thin layer of buffer is shown; the headgroups of the two lipid leaflets are resolved as two dark stripes. The distance between lipid bilayers cross-linked by C2AB is constant and ∼9 nm. (b) In control reactions, liposomes retain their rounded shape despite being brought together by chance upon ice formation. Top; a typical image of liposomes in the absence of calcium and protein. Bottom; liposomes after C2AB addition, but in the absence of calcium. The scale bar represents 50 nm.

Membrane apposition is a natural requirement for membrane fusion and the question arises of how closely synaptotagmin C2 domains can appose membranes. To answer this question, we used cryo-electron microscopy, which preserves the native hydration of biological structures. The formation of large liposomal aggregates (Fig. 6, top panel) makes it difficult to resolve individual liposome contacts; therefore, 100-fold lower concentrations of C2AB were used compared with the negative-stain conditions. Figure 7 shows that addition of the tandem C2 domains of synaptotagmin 1 in the presence of calcium typically leads to formation of well-defined flat junctions between unilamellar liposomes (Fig. 7a). At this high magnification, the lipid bilayers are clearly resolved as two dark stripes, corresponding to the phospholipid polar headgroups, which have higher electron scattering properties than the alkyl chains. The junctions consist of two parallel membrane surfaces separated by a constant distance of ∼9 nm. Note that in the absence of C2AB or calcium, the round shape of liposomes is largely preserved even when they contact each other by chance (Fig. 7b).

## Discussion

Our results provide a molecular basis for the proposed role of multiple calcium-sensitive synaptotagmins in exocytosis. During regulated secretion, the fusion of docked secretory vesicles with the plasma membrane is triggered by rapid elevation of the intracellular concentration of calcium. Membrane fusion itself is brought about by the action of the soluble NSF attachment receptor (SNARE) proteins while synaptotagmin acts as the calcium sensor.^2,6,30^ It is generally agreed that SNARE bearing membranes must come into close contact for membrane fusion to take place.^1^ We now show that tandem C2 domains of several divergent synaptotagmins are capable of causing calcium-triggered reversible vesicle aggregation.

Among the tested isoforms, synaptotagmin 1 is the major brain synaptotagmin isoform and has been studied intensively, but the majority of these studies either employed individual C2 domains or tested unidirectional phospholipid binding.^13,31^ To the best of our knowledge, only a handful of reports have addressed the possibility of vesicle clustering. An early study of synaptotagmin 1 suggested that only C2AB can cause aggregation of chromaffin granule lipids,^16^ yet in a recent study Arac and colleagues observed that C2B alone can trigger an increase in liposomal particle size measured by dynamic light-scattering.^17^ While two-directional binding to apposing membranes by tandem C2 domains can, in principle, be anticipated, liposomal aggregation by a single C2B domain was rather unexpected. Using several complementary and direct techniques, we observed that tandem C2 domains, but neither individual C2 domain, cross-link membranes efficiently. It is possible that the dynamic light-scattering thresholds set by Arac *et al.*,^17^ small differences in the C2B constructs used,^14,17^ or the alternative purification procedures contributed to the observed disparity. Although we should wait for an independent study to resolve the C2B domain discrepancy,^17^ our data show that, whilst the C2B domain can efficiently bind liposomes in a calcium-dependent manner (Supplementary Data Fig. 1), it is unable when alone to cross-link membranes. Of note, synaptotagmin C2B, by definition, is found in the context of a tandem C2 structure and thus does not operate in isolation in the cell. Our results are important for the interpretation of recent reconstitution studies that employed synaptotagmin 1 C2AB and showed that the tandem C2 domain structure is essential for membrane fusion, whereas individual C2 domains fail to stimulate this.^29,32^

Our data show a significant flattening of the membranes between liposomes cross-linked by C2AB, with a uniform bridging distance of ∼9 nm. In fact, such flattening resembles calcium-triggered membrane cross-linking driven by annexins implicated in membrane trafficking,^33,34^ suggesting that tandem C2 domains may join two apposing membranes in a similar sequential, zipper-like manner. Arac *et al* recently analysed C2AB-driven membrane apposition using cryo-electron microscopy, but the cross-linked distances they observed were of various lengths (3–11 nm) and such cross-linked regions covered only a small part of the liposomal surface.^17^ A recent negative-stain microscopy study, on the other hand, suggested that tandem C2 domains can promote local membrane buckling, leading to tubulation of liposomes.^29^ Although some liposomal splattering was observed on our carbon-coated grads (Fig. 6, lower panel), we have not seen major tubulation of liposomal membranes in the presence of C2AB and calcium, neither in negative stain or in cryo-electron microscopy experiments. This discrepancy is likely due to the differences in lipid compositions employed. We carried out experiments with a defined ratio of brain-derived phosphatidylcholine and phosphatidylserine that is commonly used in both synaptotagmin membrane-binding assays and SNARE reconstitution fusion experiments.^15,17,22,32^ In contrast, the tubulation study employed total brain Folch fraction I lipids, which are highly enriched in negatively charged phospholipids, and naturally contain components of external brain membrane leaflets, such as gangliosides and sulfatides.^29^ Therefore, it will be of interest to define, if possible, the exact lipid components that contribute to C2AB-driven membrane tubulation.^29^

In our study, we developed and adapted several robust techniques that will be useful in future work, including the investigation of membrane-embedded full-length synaptotagmin. The simple turbidity-based method can be applied also to study membrane apposition by many other double C2 domain proteins involved in membrane traffic; for example, cytosolic rabphilins and DOC proteins.^35,36^ This method is very quick and reliable, and does not involve protein or lipid modifications. The use of DiI and DiO-labelled liposomes will be very useful in visualisation of liposomal docking and for unravelling the mechanistic aspects of vesicle fusion. The optical tweezers approach (see Supplementary Data Fig. 2 and movies) clearly demonstrates the ability of tandem C2 domains to firmly cross-link moving phospholipid vesicles. This laser trap technique can now be used for time-resolved vesicle manipulation in order to decipher the docking and fusion events governed by synaptotagmin and other C2 domain-containing proteins.

In summary, the conserved nature of phospholipid membrane cross-linking driven by *Drosophila* and rat calcium-sensitive isoforms reveals the significance of this property for synaptotagmin function, and ultimately membrane fusion itself. Our results provide a necessary step towards solving the fundamental problem of how calcium triggers neurotransmission,^37^ and will be essential for interpretation of past and future studies on the function of vesicular synaptotagmin.

## Materials and Methods

### Protein purification

The synaptotagmin constructs (glutathione-*S*-transferase-tagged) have been described (C2ABs,^9,38^ individual C2 domains^39^). Recombinant proteins were expressed overnight at 19 °C in BL21 *Escherichia coli* cells. After pelleting, the bacteria were resuspended in 0.5 M NaCl, 20 mM Hepes (pH 7.3), 4 mM DTT, 2 mM MgCl_2_, DNase I and RNase 1 (Sigma, UK) before lysis with an Emulsiflex homogeniser (Avestin, Canada). Following centrifugation for 15 min at 11,000***g***, the soluble portion of the lysate was incubated with glutathione Sepharose beads (GE Healthcare) to capture glutathione-*S*-transferase-tagged protein. The beads were then washed three times with 1 M NaCl and three times with 0.1 M NaCl in buffer A (20 mM Hepes (pH 7.3), 1 mM EDTA, 1 mM DTT), before cleavage with thrombin. Proteins were bound to a HiTrap heparin column (GE Healthcare) and eluted using a linear salt gradient from 0.1 M–2.0 M NaCl in buffer A (Supplementary Data Fig. 4a). Protein purity was checked using SDS-PAGE and staining with Coomassie brilliant blue. Excess salt was removed by dialysis against buffer A containing 100 mM NaCl. An absorbance scan was used to confirm that bacterial nucleotide contaminants were removed (Supplementary Data Fig. 4b).

### Preparation of liposomes

Chloroform solutions of brain phosphatidylcholine, phosphatidylserine, phosphatidylinositol-4,5-bisphosphate and cholesterol (Avanti Polar Lipids, US) were mixed in the stated ratios and dried under nitrogen. Hydrophobic DiO or DiI dyes (Invitrogen, UK) were added where indicated in the text (10 μg of dye per 1 mg of lipid). Dried lipids were resuspended in buffer A containing 100 mM NaCl. The resulting multilamellar vesicles were extruded with 20 passes through polycarbonate Nucleopore Track-Etch membranes (Whatman, UK) of the indicated pore-size, to produce unilamellar vesicles. Size was confirmed by dynamic light-scattering.

### Turbidity assay and dynamic light-scattering

Absorbance measurements at 350 nm were done with a Genesys 6 spectrophotometer (Thermo Electron Corporation, UK). The change in absorbance indicates a change in turbidity of the liposomal solution, corresponding to increased particle size (aggregation). Experiments were carried out in UV-compatible plastic cuvettes (Eppendorf, UK) in a total volume of 100 μl. Reactions were carried out in buffer A containing 100 mM NaCl. Free calcium concentrations were determined as described.^22^ Dynamic light-scattering measurements were done using a particle size analyser (Zetasizer NanoS, Malvern Instruments, UK) with a 4 mW He-Ne laser at a wavelength of 633 nm and at a back-scattering angle of 173°. The run time was 3 min.

### Fluorescent bilayer formation and confocal microscopy

Petri dishes (35 mm) with 10 mm microwell glass bottoms (MatTek Corporation, US) were cleaned with 2 % Hellmanex solution (Hellma, UK). To create the bilayer, 120 μl of 0.25 mg/ml green DiO liposome solution (3:1 PC/PS molar ratio, 80 nm extrusion) in buffer A containing 100 mM NaCl was added to the glass microwell. The plate was incubated for 2 h at 50 °C to promote even bilayer formation. The bilayer was then washed several times with the above buffer to remove any unbound green liposomes. Docking of 12.5 μg/ml red DiI unilamellar liposomes (3:1 PC/PS molar ratio, 600 nm extrusion) to the bilayer was observed using a Radiance Confocal system (Zeiss/Bio-Rad, UK) linked to a Nikon Eclipse TE300 fluorescence microscope equipped with an oil-immersion objective (magnification 100×; 1.3 numerical aperture). DiO fluorescence was observed using an argon laser with a 488 nm band pass filter (excitation) and a 500–560 nm filter for emission. DiI fluorescence was observed with a helium/neon laser with a 543 nm band pass excitation filter and 555–625 nm emission filter.

### Negative-stain electron microscopy

PC/PS (4:1 molar ratio) liposomes (0.3 mg/ml) were mixed with 10 μM C2AB with or without 1 mM calcium for 1 h at 20 °C. Aliquots (3 μl) were then applied to glow-discharged carbon-coated electron microscopy grids before staining with 2% (w/v) uranyl acetate. Electron micrographs were recorded with a Philips EM208S instrument using low-dose transmission electron microscopy at a primary magnification of 36,000× and an accelerating voltage of 80 kV. The digital images were obtained using a Canon MP-E 65 mm macro lens.

### Cryo-electron microscopy

C2AB was added to a solution (final concentration: 5 μg/ml) containing PC/PS (4:1molar ratio) liposomes. After incubation for 15 min, aliquots of 3–4 μl were deposited on holey carbon grids and frozen quickly in liquid ethane. Cryo-electron microscopy was performed with a Tecnai (FEI) instrument operated at 200 kV. The images were recorded with a USC1000-SSCCD camera (Gatan) at a magnification of 50,000×.

### Laser trap experiment

The optical tweezers setup was based on a standard inverted microscope (Nikon TE2000-E). The laser beam coming from a single-mode, continuous-wave Yb-doped fibre laser (IPG Photonics YLM-10, λ = 1064 nm, linearly polarised) was directed into the microscope via a telescope (magnification 0.625×) and reflected by an infra-red dichroic mirror to the objective (Nikon Plan Fluor 60×, 1.4 numerical aperture, oil immersion). The laser was focused at 10 μm above the coverslip, with a power of approximately 10 mW on the sample plane. The images were collected by a CCD camera (DVC-1412AM, pixel size 6.5 μm × 6.5 μm).
