# Supplementary material for: Cross-linking of Phospholipid Membranes is a Conserved Property of Calcium-sensitive Synaptotagmins
Source: J Mol Biol. 2008 Jun 27;380(1):42–50. doi: 10.1016/j.jmb.2008.01.084 (PMC2726287; doi:10.1016/j.jmb.2008.01.084)
Supplement: Supplementary Fig. 1 [file mmc1.pdf]

Supplementary Figure 1

Connell *et al*

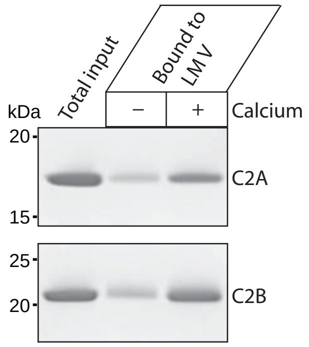

Supplementary Figure 1: Individual synaptotagmin1 C2 domains are capable of calcium-dependent phospholipid binding. Pelleting assays were carried out with large multilamellar vesicles (LMVs) as in Rickman *et al.* (2004) *Biochem J* **378,** 681-686. Briefly, dried lipids were resuspended at 2 mg/ml final concentration in 100 mM NaCl, 20 mM HEPES and 2 mM EDTA, forming LMVs. 40 µg lipid was then mixed with 3 µg of synaptotagmin1 C2A or C2B domain in the presence or absence of 1 mM free calcium. The reactions were incubated with shaking (10 min, 22°C) before centrifugation at 20 800g (10 min). The liposome pellet was washed with buffer ± calcium and bound protein analysed by SDS-PAGE followed by Coomassie staining. Both C2A and C2B bound phospholipid in a calcium-dependent manner.

Supplementary Figure 2 Connell *et al*

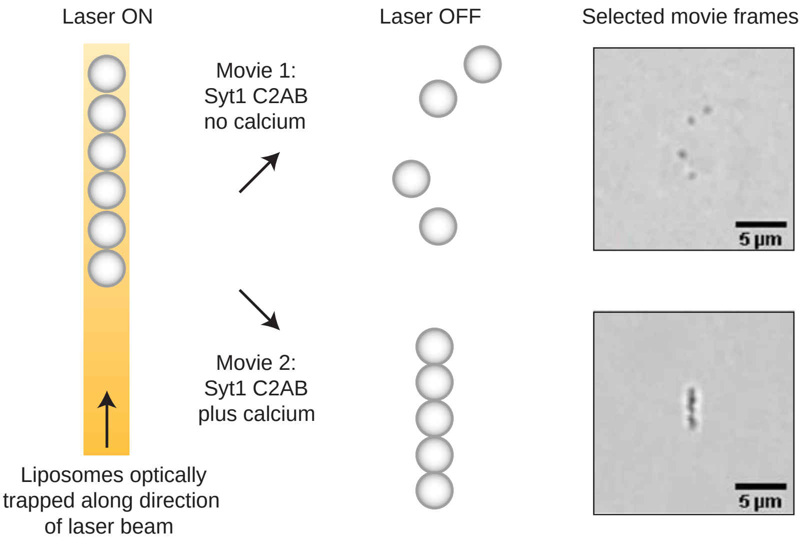

Supplementary Figure 2: Synaptotagmin 1 C2AB firmly links optically trapped liposomes. A variable number of liposomes (600 nm, 3:1 PC/PS molar ratio) were trapped in the laser beam and then the laser was switched off. Under control conditions, individual liposomes dispersed away rapidly (Movie 1), while in the presence of C2AB (2.5 µM) and calcium (1 mM) they formed stable, slowly tumbling aggregates (Movie 2). The elongated shape of the aggregates is due to the parallel alignment of trapped particles along the laser beam.

Supplementary Figure 3   Connell *et al*

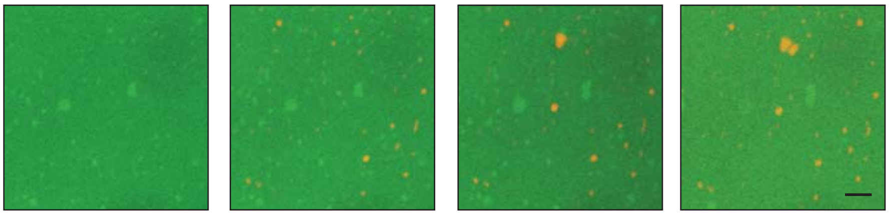

Supplementary Figure 3: C2AB promotes docking and immobilisation of liposomes onto planar membranes. Docking of red liposomes to a green planar bilayer was observed by confocal microscopy in the presence of 1 mM calcium and 2.5 µM synaptotagmin1 C2AB. Liposome docking was followed with time, and docked liposomes remained fixed in their bilayer positions, even after thirty minute incubation. Scale bar = 6 µm.

Supplementary Figure 4 Connell *et al*

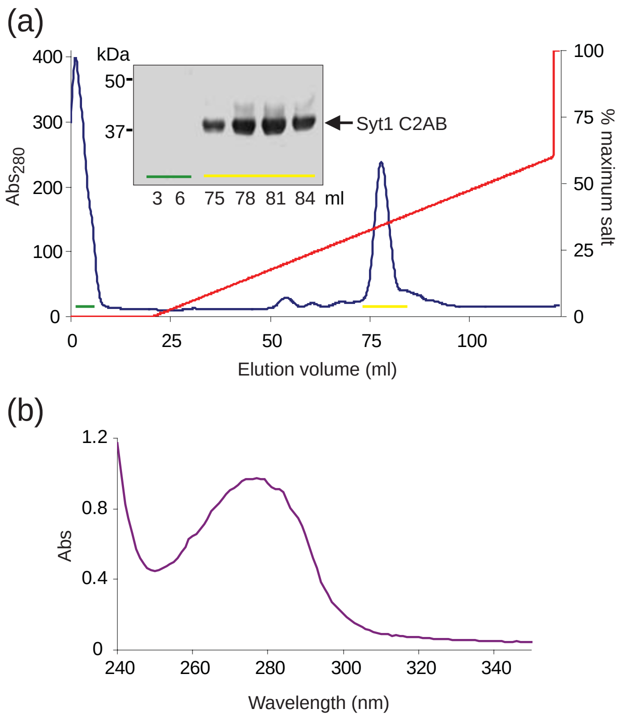

Supplementary Figure 4: Synaptotagmin C2AB purification. a) Chromatogram showing purification of C2AB on heparin column. Blue line indicates absorbance at 280 nm of column eluate. Red line indicates linear NaCl gradient used to elute the bound protein (% maximum salt). Yellow line indicates fractions containing C2AB, green line indicates DNA/RNA contaminants which do not bind to the heparin column. The Coomassie-stained SDS-gel shows lack of protein contamination. b) Absorbance scan of the eluted protein shows main peak at 280 nm (corresponding to protein absorbance) and not 260 nm (corresponding to DNA/RNA absorbance), indicating the purity of C2AB.
